# Supplementary material for: Genome-wide association study and polygenic risk prediction of hypothyroidism
Source: Nat Genet. 2025 Nov 14;57(12):3007–15. doi: 10.1038/s41588-025-02410-z (PMC12695664; doi:10.1038/s41588-025-02410-z)
Supplement: Supplementary file 1 — Supplementary Note and Figs. 1 and 2. [file 41588_2025_2410_MOESM1_ESM.pdf]

nature genetics

Supplementary information https://doi.org/10.1038/s41588-025-02410-z

Genome-wide association study and polygenic risk prediction of hypothyroidism

In the format provided by the authors and unedited

SUPPLEMENTARY INFORMATION

Genome-wide association study and polygenic risk prediction of hypothyroidism

By Rand S. et al.

Table of content

Supplementary Note

Copenhagen Hospital Biobank and Danish Blood Donor Study Page 2-3

The UK Biobank Page 3-4

FinnGen Page 4-5

The Estonian Biobank Page 5-6

23andMe, Inc Page 6-7

deCODE genetics Page 7-8

Danish General Suburban Population Study Page 8-9

Supplementary Figures

Supplementary Fig.1 Page 10

Supplementary Fig.2 Page 11

References Page 12

SUPPLEMENTARY NOTE

Cohort information, case and control definitions, ethics and details on genotyping and imputation.

Copenhagen Hospital Biobank within Chronic Inflammatory Diseases (CHB-CID) and Danish Blood Donor Study (DBDS; CHB-CID/DBDS).

Copenhagen Hospital Biobank (CHB) is a population-based biobank which encompasses data for nearly 360,000 genome-wide genotyped individuals. The Chronic Inflammatory Disease sub-cohort of the CHB was used in the present study. Samples were collected under admission to general hospitals in the capital area of Denmark between 2009 and 2020.[1] DBDS is a population-based cohort including over 160,000 Danish blood donors, of whom 120,000 were genotyped and used in the present study.[2]

Case and control definition. Cases were defined using International Classification of Disease (ICD)-10 codes: E03.8('Other specified hypothyroidism'), E03.9 ('Hypothyroidism, unspecified'), and E06.3 ('Autoimmune thyroiditis'/'Hashimoto's thyroiditis') or using the following Anatomical Therapeutic Chemical (ATC) code: H03A. Individuals with ICD-10 E05[0-9] were excluded from the analysis. Controls were remaining individuals who were free of the following ICD-10 codes: E01, E02, E03.0, E03.1, E03.2, E03.3, E03.4, E03.5, E04, E05, E06 and E07.

Thyroid hormone analysis: We used the first non-missing sample value that was within the reference range. The results of individual thyroid function tests were inverse normalized. Individuals who were either on thyroid drugs or had undergone thyroid surgery prior to the thyroid function tests were excluded, and we captured thyroid hormones using NPU codes, drugs using ATC codes, and surgical procedures using procedure codes (**Supplementary Table 24**). TSH and fT4 samples were measured on assays from Cobas (Roche Diagnostics), Alinity (Abbot Laboratories), Centaur XPT (Siemens Advia), and Dimension Vista 1500 (Siemens) from different laboratories in Denmark. All laboratories in Denmark are accredited by DANAK (following European standard ISO 15189), which ensures that all instruments follow the same procedures for quality control.

Ethics. Since biological samples stored in CHB are residual material from routine blood analyses, patients were not asked for informed consent before inclusion. Instead, information about the option to opt out are provided. A national Register on Tissue Application (Vævsanvendelsesregistret) was established in 2004 and lists individuals who opted out. Individuals listed in this register were excluded. In DBDS, all individuals provided informed consent. CHB-CID was approved by the Regional Ethical Committee in the Capital Region (H-22021178) and DBDS was approved by the National Committee on Health Research Ethics (NVC 1700407) and the Danish Data Protection Agency (P-2022-876 and P-2019-99).

Genotyping and imputation. Genotyping of samples from a total of 276,114 individuals of Danish ancestry was done using Illumina Global Screening Array chips. In sample-wise QC, samples with call rate < 97%, ambiguous gender, genetic ancestry outliers and heterozygosity outliers (>3SD) were removed. In marker-wise QC, SNPs with genotype call rate < 99%, low Hardy-Weinberg equilibrium ($< 1 \times 10^{-4}$), and MAF < 0.1% were removed. Genotypes were then long-range phased with approximately 238,000 genotyped samples from North-western Europe using Eagle. A haplotype reference panel was constructed by phasing whole-genome sequence genotypes of 15,576 individuals from Scandinavia, the Netherlands, and Ireland. Genotypes were called using Graphtyper and were subsequently imputed into the phased chip data. deCODE genetics executed all procedures which generated the data used in this analysis, including whole-genome sequencing, chip-typing, quality control, long-range phasing, and imputation.

UK Biobank (UKB).

The UKB is a large, longitudinal cohort study with more than 500,000 volunteering residents of the United Kingdom. Participants were between 40 and 69 years of age at time of recruitment (2006-2010).[3] The UKB data resource was accessed under Application ID 43247.

Case and control definition. We defined cases using the following ICD-10 codes: E03.8/E03.9/E06.3 using electronic health records. Additional cases included individuals reporting hypothyroidism at baseline or using thyroid hormone medication. In the Data-Field 20003, the following codes indicated use of thyroid hormone medication; 1140874844 (tertroxin 20mcg tablet), 1140874852 (thyroxine sodium), 140884516 (thyroxine product), 1140910814 (sodium thyroxine), 1141191044 (levothyroxine sodium), 1140909904 (tri-iodothyronine product),

1140910518 (t3 – liothyronine), and 1140910520 (sodium liothyronine). Individuals diagnosed with ICD-10 E05[0-9] were excluded from the analysis. Controls were defined as free of the following ICD-10 codes: E01, E02, E03.0, E03.1, E03.2, E03.3, E03.4, E03.5, E04, E05, E06 and E07. All participants were of White European ancestry.

Thyroid hormone analysis: In UKB primary care data covering ~ 245,000 participants, we used the first non-missing sample value that was within the reference range. The results of individual thyroid function tests were inverse normalized. Individuals who were either on thyroid drugs or had undergone thyroid surgery prior to the thyroid function tests were excluded, and we captured thyroid hormones using Read2/Read3 codes, drugs using dm+d codes, and surgical procedures using OPCS-4 codes (**Supplementary Table 24**).

Ethics. The UK Biobank cohort study received ethical approval from the Northwest Multicenter Research Ethics Committee, UK (Ref: 16/NW/0274), and all study participants provided written informed consent to participate in the study.

Genotyping and imputation. Genotype data were available for 488,380 individuals and were imputed to the HRC, UK10K and 1,000 Genomes Phase 3 reference panels using IMPUTE4 to identify ≈ 93M variants for 487,409 individuals. Using the genotyped SNPs, persons were excluded if they had high levels of missingness or heterozygosity, SNP genotype call rate < 98%, or phenotypic and genotypic gender mismatch. Further details are documented elsewhere.[4]

FinnGen Freeze 10.

FinnGen is a large public-private collaboration, which launched in 2017 and combines genome-information with digital health care data for over 500,000 individuals. FinnGen unites Finnish universities, hospitals and hospital districts, various biobanks, and international pharmaceutical companies.[5]

Case and control definition. The summary statistics from FinnGen Freeze 10 was obtained from https://www.finngen.fi/en/access_results under the identifier *"E4_HYTHY_AI_STRICT"*. Cases were defined using the following ICD-10 codes: E03.80, E03.82, E03.89, E03.9, E03.9+F02.89, E03.9+G13.2, E03.9+M14.5, the following ICD-9 codes: 244[8-9], and the following ATC code: H03A. Controls excluded the following ICD-10 codes: E01, E02, E03.0, E03.1, E03.2, E03.3, E03.4, E03.5, E04, E05, E06 and E07.

Ethics. The individuals in the FinnGen cohort provided informed consent for biobank research in accordance with the Finnish Biobank Act. Separate research cohorts, gathered before the Finnish Biobank Act was initialized (in September 2013) and start of FinnGen (August 2017), were collected based on study-specific consents and later assigned to the Finnish biobanks after approval by Fimea, the National Supervisory Authority for Welfare and Health. The Coordinating Ethics Committee of the Hospital District of Helsinki and Uusimaa (HUS) approved the FinnGen study protocol Nr HUS/990/2017. The FinnGen study is approved by Finnish Institute for Health and Welfare (THL), approval number THL/2031/6.02.00/2017, amendments THL/1101/5.05.00/2017, THL/341/6.02.00/2018, THL/2222/6.02.00/2018, THL/283/6.02.00/2019, THL/1721/5.05.00/2019, Digital and population data service agency VRK43431/2017-3, VRK/6909/2018-3, VRK/4415/2019-3 the Social Insurance Institution (KELA) KELA 58/522/2017, KELA 131/522/2018, KELA 70/522/2019, KELA 98/522/2019, and Statistics Finland TK-53-1041-17.[5]

Genotyping and imputation. Genotyping was done on Illumina and Affymetrix assays. For Illumina data, genotype calls were made GenCall and zCall algorithms, and for Affymetrix genotype calls were made with the AxiomGT1 algorithm. Individuals with ambiguous gender, high genotype missingness (>5%), excess heterozygosity (±4 SD), and non-Finnish ancestry were excluded. Variants with high missingness (>2%), low Hardy-Weinberg equilibrium ($< 1 \times 10^{-6}$), and minor allele count (<3) were excluded. Genotyped samples were pre-phased with Eagle. High coverage (25–30×) WGS of 3,775 Finnish individuals were used to develop the Finnish population-specific SISu v3 imputation reference panel with Beagle 4.1.[6]

The Estonian Genome Project (Estonian Biobank).

The Estonian Genome Project is a population-based biobank established in 2000 with more than 200,000 genotyped on genome-wide arrays mainly reflecting the age, sex, and geographical distribution of the adult Estonian population. Participants are randomly recruited by general practitioners and physicians in hospitals.[7]

Case and control definition. Cases were defined using ICD-10 codes: E03.8/E03.9/E06.3 or using ATC code H03A. Individuals with ICD-10 E05[0-9] were excluded from the analysis. Controls were remaining individuals who were free of the following ICD-10 codes: E01, E02, E03.0, E03.1, E03.2, E03.3, E03.4, E03.5, E04, E05, E06 and E07.

Ethics. Activities in Estonian Biobank follow the procedures established by the Estonian Human Genes Research Act (HGRA). All participants have signed a broad consent form, and the study is approved by the Estonian Committee on Bioethics and Human Research.

Genotyping and imputation. Genotyping was done on the Illumina GSA Microchip, which contains more than 700,000 SNP markers and population-specific markers as well.

In sample-wise QC, samples with call rate < 98%, ambiguous gender, heterozygosity outliers (> 3SD), and outliers from the European descent based on a multidimensional scaling (MDS) plot in comparison with 210 HapMap reference samples were removed. In marker-wise QC, SNPs with genotype call rate < 99%, low Hardy-Weinberg equilibrium ($< 1 \times 10^{-4}$), and MAF < 0.1% were removed. A haplotype reference panel was based on an Estonian population specific imputation reference panel from whole-genome sequencing of 2,297 Estonians.

23andMe, Inc.

23andMe is private biotech and personal genomics company that offers consumer genetic testing, which engages in collaboration with academic institutions.

Case and control definition. Definitions were based on self-reported data. Cases were individuals who answered confirmatively to one of following questions:

i) Being diagnosed with hypothyroidism or Hashimoto's thyroiditis or
ii) Currently taking medication for hypothyroidism or
iii) Having elevated thyroid stimulating hormone levels

Controls answered none of the above confirmatively and at least one negatively. Exclusion from the analysis included individuals who answered affirmatively to having:

i) Hyperthyroidism or
ii) Thyroid cancer or
iii) Received radioactive iodine treatment or
iv) Received thyroidectomy.

Ethics. All individuals included in the analysis provided informed consent and answered surveys online according to 23andMe's human subjects' protocol, which was reviewed and approved by Salus IRB (formerly Ethical and Independent Review Services), an independent external AAHRPP-accredited Institutional Review Board (IRB).

Genotyping and imputation. Samples were genotyped using four different platforms (V1-4). The V1 and V2 platforms are based on the Illumina HumanHap550+ BeadChip, which includes approximately 560,000 SNPs. The V3 platform was based on the Illumina OmniExpress+ BeadChip with around 950,000 SNPs, and the V4 platform is a custom array focusing on lower frequency coding variants including approximately 570,000 SNPs. Samples with less than 98.5% call rate were re-analyzed. In marker-wise QC, SNPs with genotype call rate < 95%, low Hardy-Weinberg equilibrium ($< 1 \times 10^{-20}$), and MAF < 0.1% were removed. The March 2012 release of the 1000 Genomes reference haplotypes was used for imputation. Phasing was performed with Beagle4 (version 3.3.1). Finally, each phased segment was imputed against the all-ethnicity 1000 Genomes haplotypes (excluding monomorphic and singleton sites) using Minimac.[8]

deCODE genetics.

deCODE genetics is an Icelandic biopharmaceutical company which combines genome-information on more than two-thirds of the Icelandic population with genealogical data, electronic health records, and medical registries.

Case and control definition. Cases were defined using ICD-10 codes: E03.8, E03.9/E06.3, ICD-9 codes: 244[8-9], and ATC code H03A. Individuals with ICD-10 E05[0-9] were excluded from the analysis. Controls were thyroid-disease individuals free of the following ICD-10 codes: E01, E02, E03.0, E03.1, E03.2, E03.3, E03.4, E03.5, E04, E05, E06 and E07. The data was extracted from electronic health records from the Hospital Discharge Register at Landspitali the National University Hospital and Register of Primary Health Care Contacts, Register of Contacts with Medical Specialists in Private Practice and Causes of Death Register (stored at the Icelandic Directorate of Health).

Ethics. The study was approved by the National Bioethics Committee (VSN-16-042, VSN-15-023) and the Icelandic Data Protection Authority. Written informed consent was obtained from all participants who donated blood samples to research. All sample identifiers were encrypted in accordance with the regulations of the Icelandic Data Protection Authority.

Genotyping and imputation. Genotyping was done Illumina HumanHap300, HumanCNV370, HumanHap610, HumanHap1M, HumanHap660, Omni-1, Omni 2.5 or Omni Express bead chips. Sample-level QC removed individuals with ambiguous gender, call-rate < 97% and individuals not

of Icelandic origin were. Marker-level QC excluded SNPs with call rate < 97.5% for common variants and < 99% for rare variants, low Hardy-Weinberg equilibrium (< 1×10^{-4}). The genome of the Icelandic population was characterized by conducting whole-genome sequencing of 49,708 Icelanders. This was followed by long-range phasing and imputation into 166,281 individuals genotyped with various Illumina platforms. Using genealogical data, the imputation was extended to 285,664 relatives of the genotyped individuals.

Danish General Suburban Population Study (GESUS).

The Danish General Suburban Population Study was a cross-sectional study of subjects aged > 20 years living in Næstved municipality, Denmark, which recruited 21,205 adults between 2010 and 2013. Participants underwent physical examination (including blood pressure measurement, anthropometric measurements, lung function assessment, 12-lead electrocardiography etc.), had blood samples drawn and completed questionnaires at baseline.[9]

Case and control definition and association to hypothyroidism polygenic risk score. First, we defined individuals with hypothyroidism through use of ICD-10 codes E03.8/E03.9/E06.3 and ATC codes H03A. Individuals with ICD-10 E05[0-9] were excluded from the analysis. The polygenic risk score (PRS) was tested on a linear basis in 760 cases and 9,628 controls using logistic regression covarying for age, sex, and 4 PC's. Next, a subset of 5,452 genotyped participants, that were free of hypothyroidism at baseline, and had thyroid-peroxidase antibodies measurements (anti-TPO) was defined. Individuals with an anti-TPO value > 100 U/mL at baseline were considered anti-TPO positive. We then evaluated the predictive ability of four different prediction models: A bench model (consistent of age, sex, and 4 PC's), a bench model + thyroid hormones, a bench model + thyroid hormones + anti-TPO positivity, and a bench model + thyroid hormones + anti-TPO positivity + PRS (**Supplementary Table 20**). Predictive abilities were calculated in terms of area under the curve receiver operating characteristic (AUC), and differences between models were assessed DeLong's test for correlated ROC curves.

Ethics. All subjects gave written informed consent, and the study was approved by the Ethics Committee for Health Research for Region Zealand (reference number: SJ-113, SJ-114, SJ-147, SJ-278) and it approved by the Danish Data Protection Agency (REG-27-2014). Written informed consent was obtained from all participants.

Genotyping and imputation. Half (10,684/21,205) of the population from GESUS were genotyped on Illumina Infinium Global Screening Array at the Institute of Clinical Molecular Biology, Kiel University, Germany. In sample-level QC, samples with call rate < 98%, heterozygosity outliers (>3 SD) were removed. In marker-wise QC, SNPs with missingness < 98%, low Hardy-Weinberg equilibrium ($P < 10^{-6}$), and MAF < 0.1% were removed. Genotyping data were imputed onto the Haplotype Reference Consortium (HRC) panel.

SUPPLEMENTARY FIGURES

Supplementary Fig. 1: Power analysis for replication of unreported genetic variants in deCODE/Estonian Biobank. Power analysis for detection of novel hypothyroidism-associated variants across a range of minor allele frequencies (MAF, X-axis) and odds ratios (OR, Y-axis), based on a significance threshold of α = 0.00028 (0.05/179 unreported variants) indicated by the dashed red line. The replication sample included 34,835 cases and 492,149 controls. Power curves were calculated for ORs of 1.03 (blue line), 1.05 (green line), 1.08 (red line), and 1.10 (purple line) across a MAF range of 0.01 to 0.3.

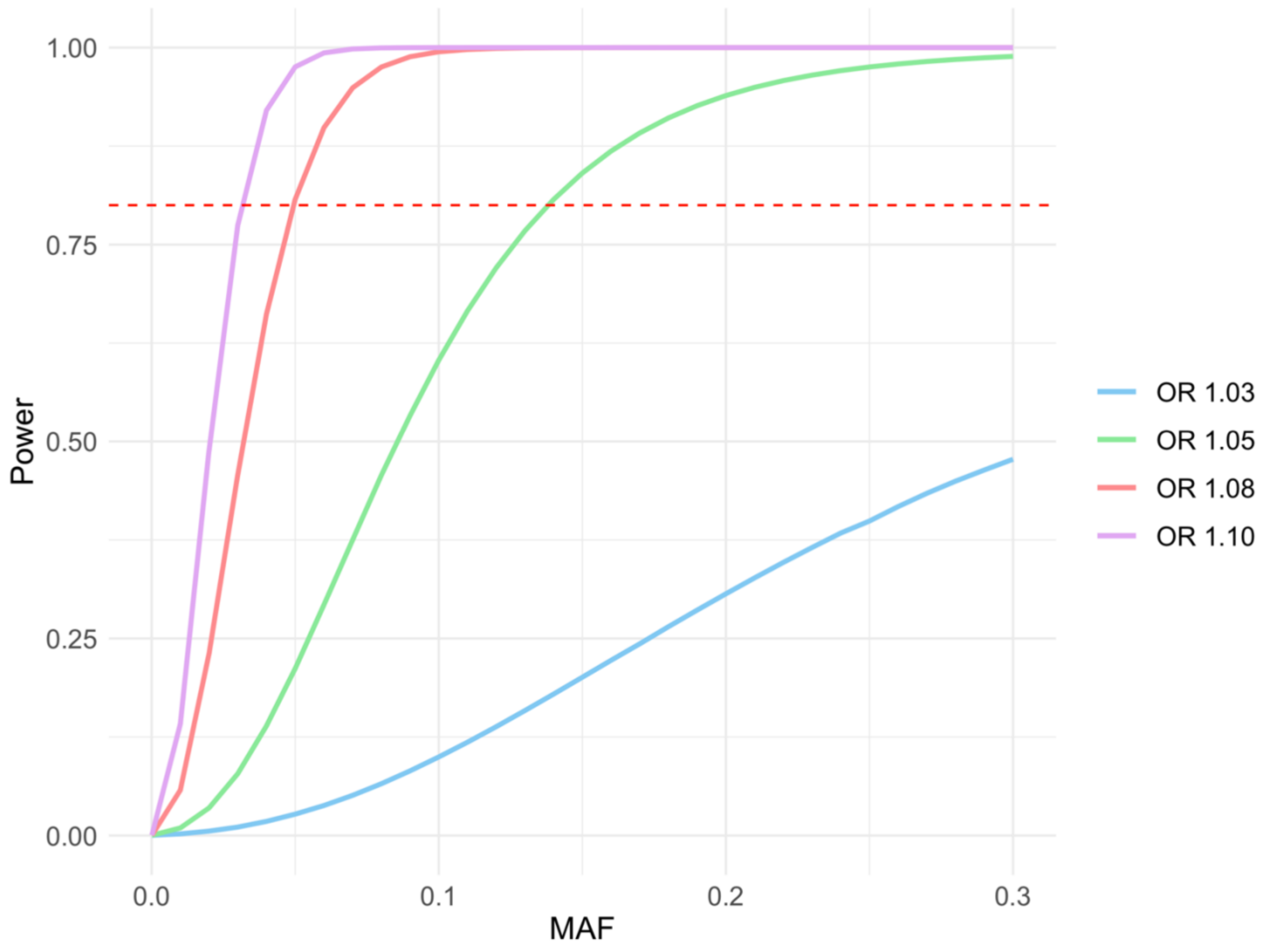

Supplementary Fig. 2: Heatmap for four hypothyroidism risk-mitigating variants with known regulatory actions in immune system function. Each cell represents the Z-score of associations between a genetic variant (Y-axis) and circulating inflammatory protein (X-axis). Asterisks denote associations passing a Bonferroni-corrected significance threshold ($P < 1.41 \times 10^{-6}$ [0.05/350 variants × 101 traits]). Cells without an asterisk but with color indicate significance below nominal significance. Shading corresponds to effect direction and strength, where blue indicates decreased levels, and red indicates increased levels of the inflammatory marker.

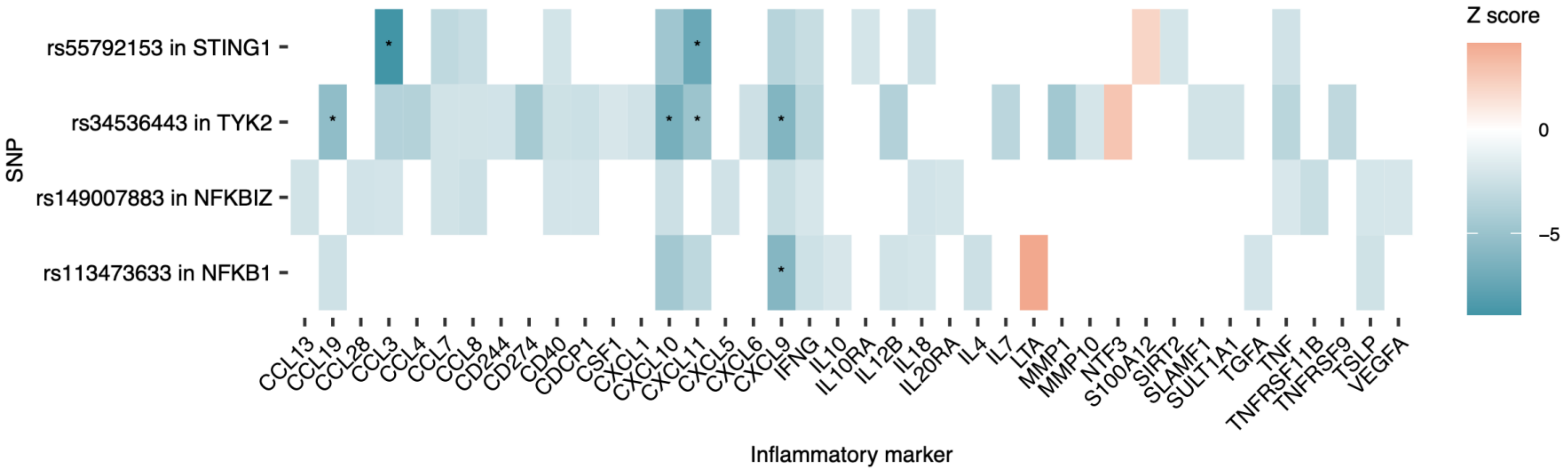

REFERENCES

1. Sørensen E, Christiansen L, Wilkowski B, et al. Data Resource Profile: The Copenhagen Hospital Biobank (CHB). *Int J Epidemiol*. 2021;50(3):719-720E. doi:10.1093/IJE/DYAA157
2. Hansen TF, Banasik K, Erikstrup C, et al. DBDS Genomic Cohort, a prospective and comprehensive resource for integrative and temporal analysis of genetic, environmental and lifestyle factors affecting health of blood donors. *BMJ Open*. 2019;9(6):e028401. doi:10.1136/BMJOPEN-2018-028401
3. Sudlow C, Gallacher J, Allen N, et al. UK Biobank: An Open Access Resource for Identifying the Causes of a Wide Range of Complex Diseases of Middle and Old Age. *PLoS Med*. 2015;12(3):e1001779. doi:10.1371/JOURNAL.PMED.1001779
4. Bycroft C, Freeman C, Petkova D, et al. The UK Biobank resource with deep phenotyping and genomic data. *Nature*. 2018;562(7726):203-209. doi:10.1038/S41586-018-0579-Z
5. Kurki MI, Karjalainen J, Palta P, et al. FinnGen: Unique genetic insights from combining isolated population and national health register data. Published online March 6, 2022. doi:10.1101/2022.03.03.22271360
6. Kurki MI, Karjalainen J, Palta P, et al. FinnGen provides genetic insights from a well-phenotyped isolated population. *Nature 2023 613:7944*. 2023;613(7944):508-518. doi:10.1038/s41586-022-05473-8
7. Leitsalu L, Haller T, Esko T, et al. Cohort Profile: Estonian Biobank of the Estonian Genome Center, University of Tartu. *Int J Epidemiol*. 2015;44(4):1137-1147. doi:10.1093/IJE/DYT268
8. Pickrell JK, Berisa T, Liu JZ, Ségurel L, Tung JY, Hinds DA. Detection and interpretation of shared genetic influences on 42 human traits. *Nature Publishing Group*. 2016;48(7). doi:10.1038/ng.3570
9. Bergholdt HKM, Bathum L, Kvetny J, et al. Study design, participation and characteristics of The Danish General Suburban Population Study. *Dan Med J*. 2013;60(A4693).
