# Supplementary material for: Genome-wide association study and polygenic risk prediction of hypothyroidism
Source: Nat Genet. 2025 Nov 14;57(12):3007–15. doi: 10.1038/s41588-025-02410-z (PMC12695664; doi:10.1038/s41588-025-02410-z)
Supplement: Supplementary file 2 — Reporting Summary [file 41588_2025_2410_MOESM2_ESM.pdf]

nature portfolio

Corresponding author(s): Dr. Søren Albertsen Rand
Dr. Jonas Ghouse

Last updated by author(s): 30th of May 2025

Reporting Summary

Nature Portfolio wishes to improve the reproducibility of the work that we publish. This form provides structure for consistency and transparency in reporting. For further information on Nature Portfolio policies, see our Editorial Policies and the Editorial Policy Checklist.

Statistics

For all statistical analyses, confirm that the following items are present in the figure legend, table legend, main text, or Methods section.

n/a	Confirmed	
☐	☒	The exact sample size (*n*) for each experimental group/condition, given as a discrete number and unit of measurement
☒	☐	A statement on whether measurements were taken from distinct samples or whether the same sample was measured repeatedly
☐	☒	The statistical test(s) used AND whether they are one- or two-sided<br>*Only common tests should be described solely by name; describe more complex techniques in the Methods section.*
☐	☒	A description of all covariates tested
☐	☒	A description of any assumptions or corrections, such as tests of normality and adjustment for multiple comparisons
☐	☒	A full description of the statistical parameters including central tendency (e.g. means) or other basic estimates (e.g. regression coefficient) AND variation (e.g. standard deviation) or associated estimates of uncertainty (e.g. confidence intervals)
☐	☒	For null hypothesis testing, the test statistic (e.g. *F*, *t*, *r*) with confidence intervals, effect sizes, degrees of freedom and *P* value noted<br>*Give P values as exact values whenever suitable.*
☒	☐	For Bayesian analysis, information on the choice of priors and Markov chain Monte Carlo settings
☒	☐	For hierarchical and complex designs, identification of the appropriate level for tests and full reporting of outcomes
☐	☒	Estimates of effect sizes (e.g. Cohen's *d*, Pearson's *r*), indicating how they were calculated

Our web collection on statistics for biologists contains articles on many of the points above.

Software and code

Policy information about availability of computer code

Data collection

No software was used to collect this data.

Data analysis

The following softwares and packages were used for data analyses:
PLINK 1.9 (https://www.cog-genomics.org/plink/1.9/)
PLINK 2.0 (https://www.cog-genomics.org/plink/2.0/)
METAL v2011-03-25 (https://genome.sph.umich.edu/wiki/METAL_Documentation)
LDAK v2023-07-01 (https://dougspeed.com/)
PoPS v0.2 (https:/github.com/Finucanelab/pops)
FUSION v2022/02/01 (http://gusevlab.org/projects/fusion/)
COLOC v5.2.3 (https://cran.r-project.org/web/packages/coloc/index.html)
LD Score Regression vl .0.1 (https://github.com/bulik/ldsc)
PRS-CS v2021-06-04 (https://github.com/getian107 /PRScs)
REGENIE v2.0.l (https://rgcgithub.github.io/regenie/)
R v4.2.2 (https://www.r-project.org/)
MungeSumstats v1.8.0 (https://www.bioconductor.org/packages/release/bioc/html/MungeSumstats.html)
pROC (https://www.rdocumentation.org/packages/pROC/versions/1.18.5)
clusterProfiler v3.21 (https://bioconductor.org/packages/release/bioc/html/clusterProfiler.html)
survival v3.6.4 (https://cran.r-project.org/web/packages/survival/index.html)
ggplot2 (https://cran.r-project.org/web/packages/ggplot2/index.html)
OpenTargets Variant2Gene v1.1 (https://genetics-docs.opentargets.org/our-approach/data-pipeline)
MendelVar v05/Dec/2023 (https://mendelvar.mrcieu.ac.uk/)

Variant Effect Predictor v111 (https://useast.ensembl.org/Tools/VEP)

For manuscripts utilizing custom algorithms or software that are central to the research but not yet described in published literature, software must be made available to editors and reviewers. We strongly encourage code deposition in a community repository (e.g. GitHub). See the Nature Portfolio guidelines for submitting code & software for further information.

Data

Policy information about availability of data

All manuscripts must include a data availability statement. This statement should provide the following information, where applicable:

- Accession codes, unique identifiers, or web links for publicly available datasets
- A description of any restrictions on data availability
- For clinical datasets or third party data, please ensure that the statement adheres to our policy

GWAS summary statistics from the meta-analysis of hypothyroidism (excluding 23andMe), TSH and T4 are publicly available at the GWAS Catalog under accession IDs: GCST90572791, GCST90572789 and GCST90572790 (https://www.ebi.ac.uk/gwas/). The corresponding hypothyroidism polygenic risk score (excluding 23andMe) is available at the PGS Catalog under accession ID: PGS005218 (https://www.pgscatalog.org/).The full GWAS summary statistics for the 23andMe discovery data set will be made available through 23andMe to qualified researchers under an agreement with 23andMe that protects the privacy of the 23andMe participants. Please visit https://research.23andme.com/collaborate/#dataset-access/. UK Biobank individual-level data are accessible upon application via the UK Biobank (https://www.ukbiobank.ac.uk/). FinnGen summary statistics are publicly available following registration at: https://www.finngen.fi/en/access_results. Data from the UK Biobank Pharma Proteomics Project (UKB-PPP) are available through Synapse (https://www.synapse.org/#!Synapse:syn51365301). GTEx v8 eQTL data can be accessed at: https://gtexportal.org/home/datasets. Individual-level data are not publicly available due to restrictions imposed by participant consent and local ethics review boards.

Research involving human participants, their data, or biological material

Policy information about studies with human participants or human data. See also policy information about sex, gender (identity/presentation), and sexual orientation and race, ethnicity and racism.

Reporting on sex and gender	The manuscript uses the term sex when referring to biological attribute, and was determined using genetic sex where available. Sex was included as a covariate in all multivariate analyses.
Reporting on race, ethnicity, or other socially relevant groupings	The included studies were exclusively individuals of European ancestry. By reducing genetic variability and confounding factors that could arise from population stratification, we aimed to enhance our ability to detect true genetic associations with the phenotypes of interest. Our study's focus on Europeans is not meant to diminish the genetic diversity and significance of other populations but was a methodological decision based on the specific aims and context of our research.
Population characteristics	Population characteristics include age, sex, ancestry, and genetic principal components for all individuals. Details on population characteristics are provided in Supplementary Tables 1 and 23, in the Online Methods section, and in the Supplementary Note.
Recruitment	Recruitment information is provided in Supplementary Note.
Ethics oversight	All human research was approved within each contributing study by the relevant institutional review board (CHB-CID/DBDS: National Committee on Health Research Ethics; deCODE: National Bioethics Committee; UKB: Northwest Multicenter Research Ethics Committee; FinnGen: The Coordinating Ethics Committee of the Hospital District of Helsinki and Uusimaa; Estonian Biobank: Estonian Committee on Bioethics and Human Research; 23andMe, Inc: Salus IRB (formerly Ethical and Independent Review Services) and the independent and external AAHRPP-accredited Institutional Review Board (IRB); GESUS: Ethics Committee for Health Research for Region Zealand) and conducted according to the Declaration of Helsinki. All participants provided written informed consent, except for CHB-CID, where patients were informed about the opt-out possibility of having their biological specimens excluded from use in research. Since 2004, a national Register on Tissue Application (Vævsanvendelsesregistret) lists all individuals who have chosen to opt out and whose samples cannot be used for research purposes. Before initiating this study, individuals listed in the Register on Tissue Application were excluded. Additional information is provided in Supplementary Note.

Note that full information on the approval of the study protocol must also be provided in the manuscript.

Field-specific reporting

Please select the one below that is the best fit for your research. If you are not sure, read the appropriate sections before making your selection.

☒ Life sciences ☐ Behavioural & social sciences ☐ Ecological, evolutionary & environmental sciences

For a reference copy of the document with all sections, see nature.com/documents/nr-reporting-summary-flat.pdf

Life sciences study design

All studies must disclose on these points even when the disclosure is negative.

Sample size	No formal statistical sample size calculations were performed prior to the study. Instead, we included all available individuals from each

Sample size	contributing cohort who met the inclusion criteria and passed quality control procedures. This approach maximizes statistical power for genetic discovery. The final sample sizes are comparable to or larger than those used in previous well-powered genome-wide association studies (GWAS) for related traits. Detailed information on sample inclusion and exclusion is provided in the Supplementary Note.
Data exclusions	Within each study, samples were excluded on the basis of sample level quality control and variant level quality control. These procedures ensure the removal of poor quality genotypes, SNPs and samples. The quality filtering steps are provided in Supplementary Table 23 and in Supplementary Note.
Replication	All GWAS analyses were conducted independently within each cohort and subsequently meta-analyzed. The primary findings were replicated in an independent meta-analysis including non-overlapping cohorts. Prior to replication, we performed power calculations to determine our ability to detect novel hypothyroidism-associated variants at various minor allele frequencies (MAFs) and odds ratios (ORs), using a Bonferroni-corrected significance threshold of $\alpha = 0.00028$ (0.05/179 replication attempts). As shown in Supplementary Fig. 1, we had >80% power to detect variants with OR ≥1.08 and MAF >0.02. Details of replication are documented in the manuscript and Supplementary Fig. 1 and Supplementary Table 8, 9, and 10.
Randomization	Randomization was not applicable to this study because it is an observational genetic association study based on pre-existing biobank data. Participants were not assigned to groups or interventions; instead, genetic and phenotypic data were analyzed as collected. The goal was to identify naturally occurring genetic variants associated with hypothyroidism and related traits, which does not require or permit random allocation.
Blinding	Blinding was not relevant to this study because it was an observational analysis of existing genotype and phenotype data from biobanks. No interventions were administered, and no subjective assessments were performed by investigators. Genotyping, quality control, and association analyses were conducted using automated pipelines without investigator influence on group assignment or outcome assessment. Therefore, the risk of bias typically mitigated by blinding was not present in this study design.

Reporting for specific materials, systems and methods

We require information from authors about some types of materials, experimental systems and methods used in many studies. Here, indicate whether each material, system or method listed is relevant to your study. If you are not sure if a list item applies to your research, read the appropriate section before selecting a response.

Materials & experimental systems

n/a	Involved in the study
☒	☐ Antibodies
☒	☐ Eukaryotic cell lines
☒	☐ Palaeontology and archaeology
☒	☐ Animals and other organisms
☒	☐ Clinical data
☒	☐ Dual use research of concern
☒	☐ Plants

Methods

n/a	Involved in the study
☒	☐ ChIP-seq
☒	☐ Flow cytometry
☒	☐ MRI-based neuroimaging

Plants

Seed stocks	*Report on the source of all seed stocks or other plant material used. If applicable, state the seed stock centre and catalogue number. If plant specimens were collected from the field, describe the collection location, date and sampling procedures.*
Novel plant genotypes	*Describe the methods by which all novel plant genotypes were produced. This includes those generated by transgenic approaches, gene editing, chemical/radiation-based mutagenesis and hybridization. For transgenic lines, describe the transformation method, the number of independent lines analyzed and the generation upon which experiments were performed. For gene-edited lines, describe the editor used, the endogenous sequence targeted for editing, the targeting guide RNA sequence (if applicable) and how the editor was applied.*
Authentication	*Describe any authentication procedures for each seed stock used or novel genotype generated. Describe any experiments used to assess the effect of a mutation and, where applicable, how potential secondary effects (e.g. second site T-DNA insertions, mosiacism, off-target gene editing) were examined.*
